# Hyperphagia of female UCP1-deficient mice blunts anti-obesity effects of FGF21

**DOI:** 10.1038/s41598-023-37264-0

**Published:** 2023-06-24

**Authors:** Marlou Klein Hazebroek, Rutger Laterveer, Maria Kutschke, Vida Ramšak Marčeta, Clarissa S. Barthem, Susanne Keipert

**Affiliations:** Department of Molecular Biosciences, The Wenner-Gren Institute, Stockholm University, 106 91 Stockholm, Sweden

**Keywords:** Obesity, Obesity

## Abstract

Increasing energy expenditure through uncoupling protein 1 (UCP1) activity in thermogenic adipose tissue is widely investigated to correct diet-induced obesity (DIO). Paradoxically, UCP1-deficient male mice are resistant to DIO at room temperature. Recently, we uncovered a key role for fibroblast growth factor 21 (FGF21), a promising drug target for treatment of metabolic disease, in this phenomenon. As the metabolic action of FGF21 is so far understudied in females, we aim to investigate potential sexual dimorphisms. Here, we confirm that male UCP1 KO mice display resistance to DIO in mild cold, without significant changes in metabolic parameters. Surprisingly, females gained the same amount of body fat as WT controls. Molecular regulation was similar between UCP1 KO males and females, with an upregulation of serum FGF21, coinciding with beiging of inguinal white adipose tissue and induced lipid metabolism. While energy expenditure did not display significant differences, UCP1 KO females significantly increased their food intake. Altogether, our results indicate that hyperphagia is likely counteracting the beneficial effects of FGF21 in female mice. This underlines the importance of sex-specific studies in (pre)clinical research for personalized drug development.

The prevalence of obesity and its comorbidities, *e.g.* type 2 diabetes, has been rising in the last few decades, posing major health and societal problems worldwide^1^. Many factors like lifestyle, hormone imbalances, and (epi)genetics have been associated with the development of obesity, but strategies to prevent and cure it have mostly been inefficient^2^. Therefore, the search for new and more effective alternatives continues.

A frequently proposed strategy to counteract obesity is the activation of brown adipose tissue (BAT). BAT contains uncoupling protein 1 (UCP1) that can dissipate energy as heat when activated^3^, leading to higher energy expenditure. Therefore, it is unsurprising that the UCP1 knockout (KO) mouse is a commonly used model to investigate possible anti-obesity strategies. However, the model presents a paradoxical and unexplained resistance to diet-induced obesity (DIO) at 20 °C^4^. The exact mechanisms underlying this resistance to DIO have long been a mystery. Previously, others described alternative thermogenic pathways, including creatine and calcium futile cycling, as a potential energy-wasting process in adipose tissue of UCP1-deficient mice^5–7^. Recently, we found that fibroblast growth factor 21 (FGF21) fully mediates DIO resistance in male UCP1 KO mice kept at mild cold^8^.

FGF21 is a hormone mainly synthesized in the liver^9^ and it is upregulated in situations of cellular and metabolic stress^10,11^. For instance, in UCP1 KO mice, BAT becomes a significant source of FGF21 at temperatures below thermoneutrality^12^. Overall, it is an important endocrine player in the regulation of energy balance as it can act on many different peripheral tissues. Its capability to improve the metabolic profile of obese subjects has increased interest in FGF21 as a therapeutic agent against metabolic diseases. For instance, pharmacological administration of FGF21 or its analogues decreases adiposity and serum lipids levels, and improves glucose homeostasis^13–16^. Besides its potential function as an anti-obesity drug, FGF21 is suggested to play a role in thermoregulation. For example, circulating FGF21 levels are increased upon cold exposure, both in rodent models (reviewed in^17^), as well as in humans^18–20^. Further, FGF21 administration increases rates of adipose tissue browning and energy expenditure^13,21,22^, which is regulated via PGC1a in WAT^23^. To date, there are conflicting data about the origin of endogenous FGF21 that is responsible for the regulation of thermogenic capacity in adipose tissue. While some state it comes solely from the liver^24^, others suggest that adipose tissue-produced FGF21 plays an autocrine role and circulating levels are dispensable^25^. However, the beneficial metabolic effects of FGF21 are not merely dependent on active brown or beige adipose tissue, and can also occur in an UCP1-independent fashion^26,27^.

Indeed, we recently observed extensive FGF21-mediated remodelling of inguinal WAT (iWAT) towards a beige phenotype in UCP1 KO mice kept at mild cold. This coincided with the moment when body weight of wildtype (WT) and UCP1 KO animals diverged^8^, suggesting an important role for iWAT metabolism in the resistance to DIO of male UCP1 KO animals kept at mild cold. No differences in futile creatine cycling could be observed but our data hinted towards futile lipid cycling, indicated by an upregulation of genes related to lipolysis, lipogenesis as well as glyceroneogenesis^8^. However, the exact mechanisms by which FGF21 controls the resistance to DIO by iWAT remodelling are incompletely understood. Further, it is unknown whether the protective effect of FGF21 is present in female UCP1 KO mice as well, as young females are generally better protected against metabolic diseases^28^. Here, we aimed to investigate potential sexual dimorphism in the FGF21-mediated response to high-fat diet (HFD) in mild cold.

We show that the resistance to DIO is sex-specific and only occurs in UCP1 KO males but not females. Molecular regulation is similar between sexes of UCP1 KO mice, including upregulated levels of circulating FGF21, remodelling of iWAT towards a beige phenotype and induced lipid metabolism compared to WT. Strikingly, female UCP1 KO mice had a higher food intake compared to WT controls, suggesting that hyperphagia might counteract the resistance to DIO. These data underscore the importance of studying both sexes to better understand differences in cytokine action and energy balance on body weight to develop more effective weight loss strategies.

## Results

### Female UCP1 KO mice are not resistant to diet-induced obesity at mild cold despite high circulating FGF21 levels

**Figure 1:**
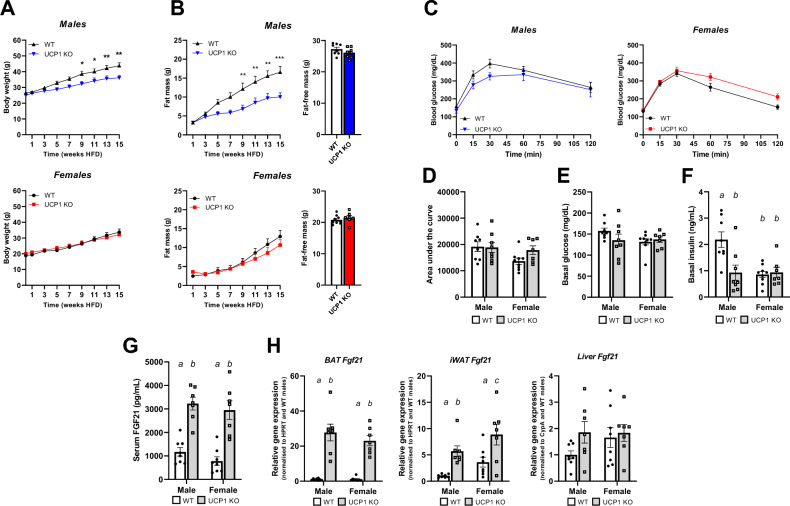
Female UCP1 KO mice are not resistant to diet-induced obesity at mild cold despite high circulating FGF21 levels. **(A)** Body weight development of male (blue/black) and female (red/black) mice on HFD for 15 weeks at 18 °C. **(B)** Fat mass development and final fat-free mass of male and female mice on HFD for 15 weeks. **(C)** Glucose tolerance test after 14 weeks of HFD feeding (after 6 h of food withdrawal) of both male and female mice and **(D)** related area-under-the-curve values. **(E)** Basal glucose **(F)** and insulin levels at T0 of the GTT after 14 weeks HFD and 6 h food withdrawal. **(G)** Circulating levels of FGF21 of male and female mice after 5 weeks of HFD feeding. **(H)** Gene expression of *Fgf21* in BAT, iWAT and liver (normalized to the male WT group). All groups represent *n* = 7–9. Significant differences are indicated with different letters (*P* < 0.05) or stars (*P < 0.05, **P < 0.01, ***P < 0.001).

To reduce the confounding effects of thermal stress, male and female mice were kept at 30 °C until the switch to HFD and mild cold. Despite using a different-based HFD (lard instead of coconut oil) and a slightly reduced ambient temperature, body weight development of male UCP1 KO mice confirmed previous results^8^, showing reduced body weight and body fat gain compared to WT littermates (Fig. 1A,B), with no difference in fat-free mass after 15 weeks of HFD feeding (Fig. 1B). Resistance to DIO was not seen in female mice, where WT and UCP1 KO gained comparable amounts of weight and body fat over time (Fig. 1A,B), reaching the same body fat content as WT males (Fig. S1A). The sexual dimorphism in body fat was reflected in adipose tissue weights, as shown by a lower iWAT and gonadal WAT (gWAT) weight of male but not female UCP1 KO mice compared to WT (Fig. S1B). BAT of UCP1 KO mice of both sexes was heavier than in WT littermates (Fig. S1B). After 14 weeks of HFD feeding, a glucose tolerance test (GTT) showed no significant differences in glucose clearance (Fig. 1C,D) or basal glucose between WT and UCP1 KO mice in both sexes (Fig. 1E). However, basal insulin levels were significantly lower in UCP1 KO males compared to WT (Fig. 1F). In females, both genotypes had comparable low insulin levels, similar to UCP1 KO males. At an earlier GTT (Fig. S1C,D), after 4 weeks of HFD, all genotypes and sexes showed comparable low levels of basal insulin (Fig. S1E), suggesting insulin sensitivity decreased only in male WT mice upon prolonged HFD feeding. Using UCP1/FGF21 double knockout mice we have previously shown that DIO resistance in male UCP1 KO mice was mediated by endogenous FGF21^8^. Thus, we next examined serum levels of FGF21, after 5 weeks of HFD feeding, where differences between the male WT and UCP1 KO first appear (Fig. 1A). As expected, we detected an upregulation of circulating FGF21 in the UCP1 KO males (Fig. 1G). Surprisingly, the same pattern was seen for the female UCP1 KO mice, indicating that although females do not show DIO resistance, the regulation of FGF21 is similar between sexes in UCP1 KO mice. Increased FGF21 levels coincided with upregulation of *Fgf21* gene expression in both BAT and iWAT, but not in liver in male and female UCP1 KO mice (Fig. 1H). Subsequently, we examined whether the expression of the FGF21 receptor complex shows sexual dimorphism, hinting to a difference in FGF21 sensitivity, but could not detect significant sex differences in any of the tissues measured (Fig. S2). No differences in body weight, body composition and serum levels of FGF21 were observed in a separate HFD fed cohort kept at 30 °C (Fig. S1F–H), indicating that a combination of HFD feeding and cold exposure is required to induce the resistance to DIO in UCP1 KO males.

### Remodelling of iWAT in UCP1 KO mice of both sexes

**Figure 2:**
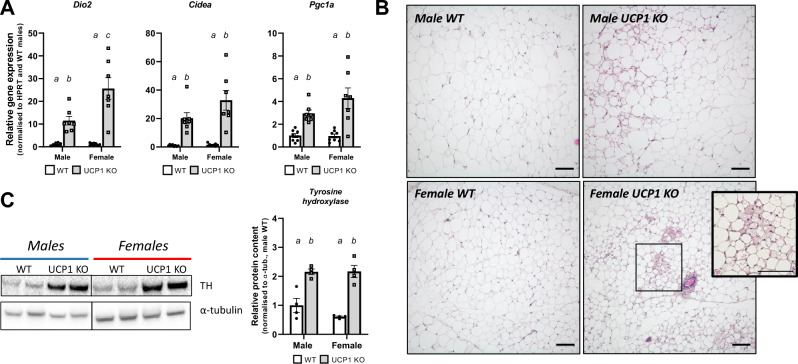
Inguinal WAT shows a beige phenotype in UCP1 KO mice of both sexes. **(A) **Gene expression of browning-related genes *Dio2, Cidea* and *Pgc1a* in male and female mice after 5 weeks of HFD (normalized to the male WT group). **(B)** Haematoxylin and Eosin staining of iWAT sections of male WT, male UCP1 KO, female WT and female UCP1 KO mice after 5 weeks of HFD. The scale bar indicates 100 µm. **(C)** Protein levels of tyrosine hydroxylase (TH) in iWAT of male and female mice after 5 weeks of HFD (normalized to the male WT group). Figure 2A represents *n* = 7–8. Figure 2B shows the best representative image of the group (of which 4 animals were analysed). Figure 2C shows two representative bands of each group (of which 4 animals were analysed). *Source* data and the selection of the blots can be found in Fig. S5. Significant differences are indicated with different letters (*P* < 0.05).

**Figure 3:**
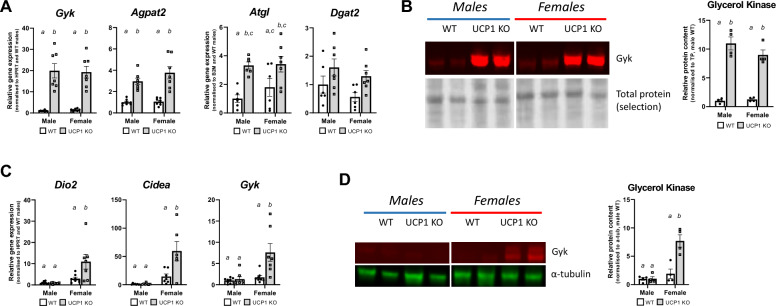
Inguinal WAT shows hints of increased lipid metabolism in male and female UCP1 KO mice, whereas gonadal WAT shows a beige phenotype in females only. **(A)** Gene expression from iWAT of genes related to lipid cycling (*Gyk, Agpat2, Atgl and Dgat2*) in male and female mice after 5 weeks of HFD (normalized to the male WT group). **(B)** Protein levels of glycerol kinase (Gyk) in iWAT of male and female mice after 5 weeks of HFD (normalized to the male WT group). **(C)** Gene expression from gWAT of genes related to browning and lipid cycling (*Dio2, Cidea and Gyk*) in male and female mice after 5 weeks of HFD (normalized to the male WT group). **(D)** Protein levels of glycerol kinase (Gyk) from gWAT of male and female mice after 5 weeks of HFD (normalized to the male WT group). Figure 3A and 3C represent *n* = 7–8. Figure 3B and D show two representative bands of each group (of which 4 animals were analysed). *Source* data and the selection of the blots can be found in Fig. S5. Significant differences are indicated with different letters (*P* < 0.05).

Previously, we have shown with RNA-sequencing that iWAT is the main tissue of interest in the FGF21-mediated regulation of resistance to DIO in male UCP1 KO mice^8^. Consistent with this, we observed browning of iWAT in UCP1 KO males, reflected by upregulation in *Dio2, Cidea,* and *Pgc1a* as well as an increased number of cells with multilocular lipid droplets compared to WT after 5 weeks of HFD, but not after 15 weeks of HFD (Figs. 2A,B, S3A). This reinforces the hypothesis that the upregulation of browning-related genes indeed goes hand in hand with morphological changes within the tissue. Browning of WAT was previously linked to resistance to DIO in different mouse models, among which the UCP1 KO model^8,29^. Remarkably, female UCP1 KO mice showed the same phenotype of increased browning of iWAT despite not being resistant to DIO. Moreover, UCP1 KO mice of both sexes displayed increased protein levels of tyrosine hydroxylase compared to WT mice suggesting a higher innervation (Fig. 2C). Previously, iWAT has been proposed to be the tissue responsible for alternative mechanisms for thermogenesis, leading to the lean phenotype in UCP1 KO males^8^. Next to the induced browning of iWAT, which is suggested to be linked to increased lipolytic activity^30,31^, we further observe an upregulation of multiple genes in lipid metabolism (Fig. 3A). We saw a strong increase in glycerol kinase (*Gyk*) expression (Fig. 3A), a gene that is usually expressed in very low quantities in white adipocytes but upregulated in beige adipocytes^32,33^. Glycerol kinase protein levels were also increased in UCP1 KO mice of both sexes (Fig. 3B). Moreover, we observed increased expression of *Agpat2* in both sexes (Fig. 3A) and *Lpin1* in females (Fig. S3B) and a tendency in *Atgl* and *Dgat2* upregulation (Fig. 3A). *Pparg* expression—previously linked to mediate BAT thermogenesis^34^-was upregulated in iWAT of both male and female UCP1 KO mice (Fig. S3B). Altogether, this could give iWAT increased capacity for lipid re-esterification, which is a potential driving force for alternative thermogenesis.

As increased lipid metabolism in WAT might be reflected in serum lipids, we next investigated circulating levels of triglycerides (TGs), glycerol and non-esterified fatty acids (NEFAs). Both male and female UCP1 KO mice have significantly lower levels of triglycerides in serum (Fig. S3C). In males, no differences were observed in circulating glycerol and NEFA levels (Fig. S3D,E). In female UCP1 KO mice, circulating glycerol levels were lower compared to WT mice, but NEFA levels were similar to those of WT littermates (Fig. S3D,E). Potentially, the changes in iWAT demand higher uptake of TGs, therefore leading to lower levels in circulation.

Besides iWAT, we investigated possible molecular changes in gWAT. In males, this tissue is known to be less innervated and to present a lower adrenergic responses compared to iWAT^35,36^, but recruitment of beige adipocytes upon beta-adrenergic stimulation has been observed in female mice^37^. In line with this, we observed an upregulation of *Dio2*, *Cidea* and *Gyk* in gWAT of female UCP1 KO animals, but not males (Fig. 3C). Protein levels of glycerol kinase were also upregulated only in UCP1 KO females and not males (Fig. 3D). Altogether, this indicates sex-specific browning of gWAT in female UCP1 KO that could coincide with increased lipid cycling capacity.

### Metabolic phenotyping displays no genotypic differences during the diet and temperature switch

**Figure 4:**
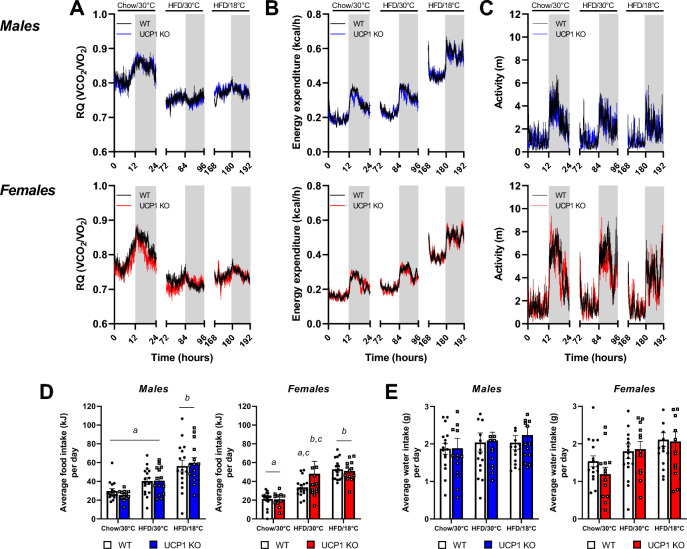
Metabolic phenotyping displays no genotypic differences during the diet and temperature switch. **(A)** Respiratory quotient (RQ) of male (black/blue) and female (black/red) mice during a period of chow feeding at 30 °C (0-24 h), HFD feeding at 30 °C (72-96 h) and HFD feeding at 18 °C (168-192 h). **(B)** Energy expenditure of male and female mice during a period of chow feeding at 30 °C, HFD feeding at 30 °C and HFD feeding at 18 °C. **(C)** Average activity levels of male and female mice during a period of chow feeding at 30 °C, HFD feeding at 30 °C and HFD feeding at 18 °C. **(D)** Average food and **(E)** water intake per day of male and female mice during a period of chow feeding at 30 °C, HFD feeding at 30 °C and HFD feeding at 18 °C. Figure 4A–C represent *n* = 15–17. Figure 4D, E represent *n* = 11–17. Significant differences are indicated with different letters (*P* < 0.05).

Our molecular characterization of adipose tissue revealed increased browning of white fat in male and female UCP1 KO mice compared to WT. However, female UCP1 KO mice are not resistant to DIO like their male counterparts, despite the increased FGF21 serum levels and beige fat. Thus, we next performed mouse metabolic phenotyping to identify the parameters of energy metabolism responsible for the differences in obesity development. We performed indirect calorimetry to measure food and water intake, energy expenditure (EE), activity and the respiratory quotient (RQ) during the diet and temperature switches. After the exchange of diet from chow to HFD, the RQ displayed a lower amplitude between light and dark phases and decreased in both genotypes and sexes due to the change in the macronutrient composition of the diets (Fig. 4A). After the temperature switch to 18 °C, the RQ slightly increased, but kept its lower amplitude. For both males and females, no differences between WT and UCP1 KO animals were observed in any of the conditions. The EE was similar between genotypes during chow feeding, went slightly up in both genotypes and sexes after the switch to HFD (Figs. 4B; S4A) and doubled when the temperature was changed to 18 °C (Figs. 4B, S4A). Again, no genotypic differences were observed for both males and females in any of the conditions. In females, activity remained similar between genotypes during all conditions (Figs. 4C, S4B). In males, total activity went down with the switch to 18 °C in WT only, and a difference was seen between WT and UCP1 KO mice during chow and HFD feeding at 30 °C (Figs. 4C, S4B). Food intake was similar between WT and UCP1 KO animals of both sexes during all conditions (Fig. S4C). However, at 18 °C food intake was increased compared to 30 °C in both males and females, leading to a higher average intake (Fig. 4D). Water intake remained similar in all conditions for males and females, and no differences were observed between WT and UCP1 KO animals (Fig. 4E). Overall, the lack of genotypic differences in energy metabolism indicates that changes in susceptibility to DIO do not occur before or during diet and temperature changes.

### Long-term high-fat diet feeding leads to an increased food intake in female UCP1 KO mice

**Figure 5:**
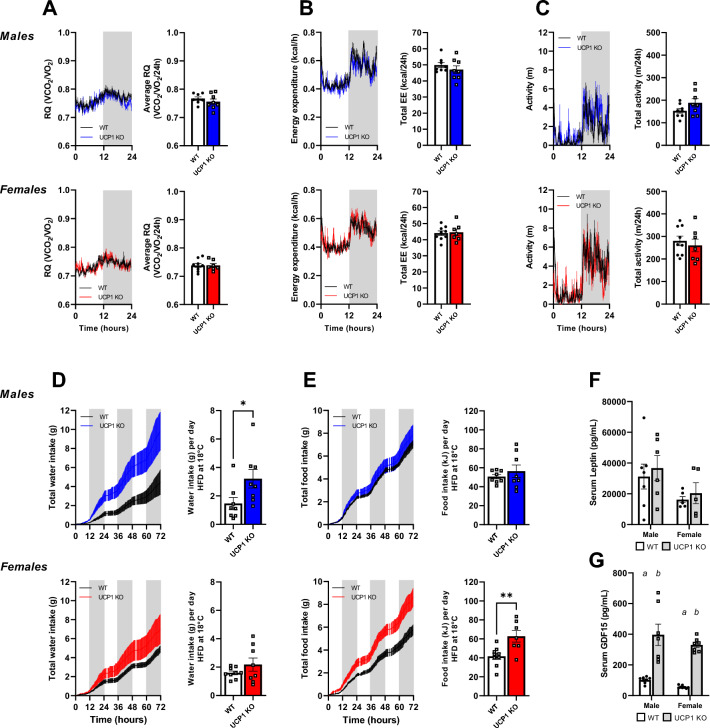
Long-term high-fat diet feeding leads to an increased food intake in female UCP1 KO mice. **(A)** Traces and average/total of 24 h RQ, **(B)** energy expenditure and **(C)** activity levels of male (black/blue) and female (black/red) during indirect calorimetry measurements at 5 weeks of HFD feeding at 18 °C. **(D)** Accumulative water intake over 72 h and average water intake per day of male and female mice. **(E)** Accumulative food intake over 72 h and average food intake per day of male and female mice. **(F)** Serum levels of leptin and **(G)** GDF15 after 5 weeks of HFD feeding. Figure 5A–E represent *n* = 7–9. Figure 5F, G represent *n* = 5–8. Significant differences are indicated with different letters (*P* < 0.05) or stars (*P < 0.05, **P < 0.01).

Next, we investigated possible changes at the time point where the body weight of male WT and UCP1 KO mice starts to diverge—after 5 weeks of HFD feeding. This corresponds with the time point of increased FGF21 levels and white fat browning (Fig. 1G and Fig. 2A,B). For both males and females, no differences between genotypes were observed regarding RQ, EE or activity (Fig. 5A–C). Water intake was higher in UCP1 KO males (Fig. 5D), as observed previously upon high circulating FGF21 levels^8^. Indeed, a higher water intake is associated with increased FGF21^38^. Interestingly, UCP1 KO female mice do not show a significant increase in water intake (Fig. 5D), suggesting high circulating FGF21 levels have less effect on water intake in female mice. Instead, they have a higher food intake compared to WT littermates, which was not observed in males (Fig. 5E). Previously, it was reported that male UCP1 KO mice have an altered food intake pattern compared to WT mice, even though total food intake was similar^39^. However, a detailed analysis of food intake patterns of our data showed no differences for the males (Fig. S4D–G). Further, it revealed that the increased food intake in females is due to increased meal size, whereas no changes were seen in the total number of meals, average meal duration or interbout time (Fig. S4D–G).

The striking difference in food intake between female WT and UCP1 KO mice, which is not accompanied by any changes in RQ, EE or activity, suggests an endocrine crosstalk, possibly via the brain. One endocrine acting factor that is well-known to regulate food intake via the brain is leptin. Circulating leptin was overall higher in the male groups compared to females, however, no differences between WT and UCP1 KO were observed (Fig. 5F). Another factor that is linked to food intake regulation is growth differentiation factor 15 (GDF15). Regulation of this protein often goes hand in hand with FGF21^11^ and can lead to reduced consumption of fat rich food^40^. Interestingly, circulating GDF15 is dramatically increased in UCP1 KO mice (Fig. 5G), in both males and females. The fact that there are no sex differences along with higher food intake in female UCP1 KO mice suggests that plasma GDF15 is not the driving force of the differences in food intake.

## Discussion

The UCP1-deficient mouse model is commonly used to investigate the role of BAT in anti-obesity strategies. However, in mild cold male UCP1 KO mice show a paradoxical resistance to DIO^4^. Previously, we reported a key role for FGF21 in this phenomenon, with remodelling of iWAT as a central feature^8^. Given the increased interest in FGF21 for drug development against metabolic diseases^41^, it is important to get broader understanding of its effects by including both sexes in (pre)clinical research.

Here, we used male and female mice to explore potential sex differences in the UCP1 KO model upon mild cold. We used a slightly colder ambient temperature (18 °C) than previously^8^ to increase circulating FGF21 levels further, with the goal of increasing effect size and determining the underlying causes of FGF21-mediated DIO resistance. We found that resistance to DIO is sustained in male UCP1 KO mice but absent in females. In line with our hypothesis, exposure to 18 °C led to increased circulating FGF21 levels in both WT and UCP1 KO males compared to our previous study at room temperature^8^. However, this upregulation did not affect the resistance to DIO, as body weight of male UCP1 KO mice developed similarly to what we reported earlier^8^. Despite the physiological differences between the sexes, regulation on the molecular level was similar between males and females, with higher circulating FGF21 levels, and increased browning and remodelling of iWAT. In females, gWAT also displayed a beige phenotype, which was not observed in males. Although no differences were observed in measurements of energy metabolism between WT and UCP1 KO males, UCP1 KO females had an increased food intake after 5 weeks of HFD. Altogether, our data indicate a similar regulation on a molecular level towards a potential source of energy-wasting via adipose tissue browning, where females might compensate leanness with a higher food intake.

One of the proposed mechanisms of alternative thermogenesis underlying the energy loss in male UCP1-deficient mice is futile lipid cycling in iWAT^8^. A key component of this process is the upregulation of glycerol kinase that happens upon cold exposure and browning of WAT^12,33,42^, giving the cell increased capacity to convert glycerol into glycerol-3-phosphate – a process that requires ATP. Previously, it was shown that increased glycerol kinase activity triggers a rise in glycerol incorporation into TG storage of cultured white adipocytes, and a reduced release of free fatty acids (FFA)^43^, suggesting induced ‘recycling’ of lipids within the cell. More recent, the use of a lipid futile cycle to maintain body temperature in mild cold by UCP1 KO mice was discovered in BAT^44^. By using^2^H^2^O-tracing to determine rates of lipid cycling, the authors showed increased rates of TG/FA cycling and decreased rates of de novo lipogenesis in UCP1-deficient BAT. Our results are in line with these studies, showing lower blood TG levels in both sexes and lower glycerol levels in UCP1 KO females. Together with the changed morphology of the iWAT—*i.e.* multilocularity at early stages and smaller adipocytes after long-term HFD—which is linked to higher rates of lipolysis^45^, these results hint towards increased futile lipid cycling in iWAT underlying the resistance to DIO observed in the males in our study.

As the differences between WT and UCP1 KO mice were clearly visible on a molecular level, it is surprising that no differences were seen in energy expenditure, specifically for DIO-resistant males. We have previously shown that small differences in energy balance can drive strong effects on fat accumulation with long-term HFD feeding^8^. We hypothesize that those small effects were not detectable in the short measurement time of our study, and extended measurement periods together with a higher n-number would be needed to overcome this issue. Further, given that the molecular regulation, and specifically that of the key player in DIO resistance—FGF21, is similar in male and female UCP1 KO mice, it is interesting that female UCP1 KO mice do not display resistance to DIO. Furthermore, unlike male mice, female UCP1 KO mice do not show a significant increase in water intake compared to WT mice, despite high circulating levels of FGF21^38^. Both findings suggest a different metabolic response to FGF21 in female compared to male UCP1 KO mice. To date, most research on FGF21 was performed on males, leaving its effects on female metabolism understudied. However, a few recent publications started uncovering sex differences upon FGF21 treatment. For instance, in A^*y*^ mice, females were resistant to weight loss and their food intake was increased upon FGF21 treatment, which was in stark contrast with the effects in males^46^. Later, Chaffin et al. showed that administration of FGF21 triggers body weight loss in male C57BL/6J, but not female mice, even though circulating FGF21 levels in plasma were similar between sexes^47^. The reduction in body weight of males was attributed to decreased fat mass, as was true for our study. Interestingly, in their study food intake was also increased in female and not male mice upon FGF21 treatment, which was fully reversed in ovariectomized and aged female mice^47^. These results strongly suggest that the effects of FGF21 administration to decrease body weight are counteracted by ovarian/sex hormones and this leads to increased food intake, as we observed as well.

Previously, others have put a focus on the relationship between high circulating FGF21 levels and sex hormones in females. Here, they find that high levels of circulating FGF21 (induced by administration or overexpression) were linked to impaired fertility in female mice^48,49^. The authors found a distinct role for FGF21 as it communicates via the hypothalamic-pituitary–gonadal axis and lead to disrupted oestrous cycles^48^. Although others are more sceptical about the role of high endogenous levels of FGF21 in this phenomenon^50^, they did observe that HFD feeding in FGF21 transgenic mice can rescue fertility by increasing hypothalamic cues to stimulate the oestrous cycle. Alongside, their FGF21 transgenic mice display hyperphagia, which suggests that female mice with high circulating FGF21 levels experience internal cues for ‘starvation’. Thus, the increased food intake observed after 5 weeks of HFD might be a compensatory response for UCP1 KO females to keep their adiposity levels equally high to WT females, and with that protecting reproductive capacity^51^. However, this possible “rescue” mechanism of female UCP1 KO mice is questionable, since high fat diet and obesity itself impairs female reproductive function^52^. Collectively, it remains unknown what is driving the increased food intake in UCP1 KO females, as we excluded a role for leptin or GDF15. Instead, an interplay between sex hormones and high endogenous FGF21 levels, with regulation via the brain, might be underlying this effect. Further investigations are required to understand this complex interplay. Even though many aspects underlying the sex differences remain elusive, data suggest that hyperphagia of female UCP1-deficient mice blunts anti-obesity effects of FGF21 induced WAT browning. These sexual dimorphisms underline the importance of including males and females in research, especially with the development of FGF21 as a therapeutic agent in mind.

## Materials and methods

### Animal study

Homozygous WT and UCP1 KO male and female mice (C57BL/6J background) derived from heterozygous breedings were included. Mice were born and raised at 24–25 °C and transferred to 30 °C at the age of 4–5 weeks to prevent adaptation to thermal stress. Mice were group-housed with ad libitum access to food (Altromin chow, 1324P, Brogaarden) and water on a 12:12 h dark–light cycle (light on at 7:00 a.m. CET). At the age of 10–12 weeks, mice were single housed and placed in a respirometry system (Sable Systems, Promethion Core System) to measure energy expenditure (EE), respiratory quotient (RQ), food and water intake, and activity. Mice were switched to a 60% high-fat diet (SAFE® U8954 Version 205, SAFE labs) after three acclimation days, and a temperature switch to 18 °C was made two days later. The day prior to diet switch was used for the analysis of parameters on chow diet at 30 °C, the day after diet switch was used for the analysis of HFD at 30 °C, and the analysis of HFD at 18 °C was performed on data from three days after the temperature switch. A second measurement period in the respirometry system took place after 5 weeks of HFD feeding. Throughout the whole experimental period, body weight was measured weekly. Body composition (by use of the EchoMRI Whole Body Composition Analyzer) was measured every other week. After 5 or 15 weeks of HFD feeding, mice were sacrificed 2–4 h after the lights went on, and serum and tissue samples were collected. Alongside the 18 °C groups, a separate cohort was kept at 30 °C with HFD feeding. This cohort was not housed in the respirometry system but underwent the same interventions as the 18 °C group and was sacrificed after 5 weeks of HFD feeding. The animal maintenance and experiments were performed under consideration of the ARRIVE guidelines and were all in accordance with the ethical regulations for animal research and approved by the Stockholm Ethics committee.

### Glucose tolerance test

After 4 and 14 weeks of dietary intervention, an intra-peritoneal (i.p.) glucose tolerance test (GTT) was performed. 30% glucose solution (1.5 mg/g of body weight) was injected i.p. after a 6-h fast. Glucose levels were measured before injection (T0) and 15, 30, 60 and 120 min after injection. At T0, a small volume of blood was taken for insulin analysis.

### Gene expression analysis

RNA extraction was performed with TriReagent (T9424, Sigma) according to the manufacturer’s instructions. cDNA synthesis was performed on 1 µg of total RNA using the QuantiTect Reverse Transcription Kit (205,314, Qiagen). The PCR mix contained cDNA (corresponding to 5 ng of RNA), gene-specific primers (Table 1) and SybrGreen Master Mix (S4438, Sigma). Gene expression was calculated with the ^ΔΔ^Ct-method using *Hprt or B2m* (BAT/WAT) and *Hprt or Cypa* (Liver) for normalization. All data are shown as values relative to the male WT group at 5 weeks of HFD.

**Table 1:** Primer sequences.

Primer name	Forward sequence	Reverse sequence
*Agpat2*	TCACCTCAGGAACAATCAAGG	AATGGCAGAGTTCTCTTGGG
*Atgl*	CAAGGGGTGCGCTCTGTGGATGG	AGGCGGTAGAGATTGCGAAGGTTG
*B2m*	CCCCACTGAGACTGATACATACGC	AGAAACTGGATTTGTAATTAAGCAGGTTC
*Cidea*	AATGGACACCGGGTAGTAAGT	CAGCCTGTATAGGTCGAAGGT
*Cypa*	ATGGTCAACCCCACCGTGT	TTTCTGCTGTCTTTGGAACTTTGTC
*Dgat2*	TGCCCTACTCCAAGCCCATCACC	TCAGTTCACCTCCAGCACCTCAGTCTC
*Dio2*	AATTATGCCTCGGAGAAGA	GGCAGTTGCCTAGTGAAAG
*Fgf21*	CTGCTGGGGGTCTACCAAG	CTGCGCCTACCACTGTTCC
*Fgfr1*	TGTTTGACCGGATCTACACACA	CTCCCACAAGAGCACTCCAA
*Gyk*	GTCACAATGGAGCGGTTTG	TTTATGGGATACCACTTTCTGGA
*Hprt*	CAGTCCCAGCGTCGTGATTA	AGCAAGTCTTTCAGTCCTGTC
*Klb*	AACCAAACACGCGGATTTC	GATGAAGAATTTCCTAAACCAGGTT
*Lpin1*	AAGAGACTGACAACGATCAGGA	TTCCCCAGAGAACCAGTGGAT
*Pgc1a*	AGCCGTGACCACTGACAACGAG	GCTGCATGGTTCTGAGTGCTAAG
*Pparg*	CTCCAAGAATACCAAAGTGCGA	GCCTGATGCTTTATCCCCACA

### Western blot

Protein extraction was done with RIPA lysis buffer (150 mM NaCl, 1% IGEPAL CA-630, 0,5% sodium deoxycholate, 0,1% SDS, 50 mM Tris, pH 8.0) containing 1 × protease/phosphatase inhibitors. Samples were lysed in the TissueLyser (Qiagen) until the tissue was fully homogenised. Samples were incubated on ice for 30 min, followed by a centrifugation step (30 min, 4 °C, 18,000 g). The supernatant was collected, and centrifuged again (20 min, 4 °C, 18,000 g). Then, supernatant was used to quantify the protein concentration with a Bradford assay (B6916, Sigma). The following primary antibodies were used: anti-tyrosine hydroxylase (ab137869, Abcam, diluted 1:2000), anti-glycerol kinase (ab126599, Abcam, diluted 1:2000) and anti-alpha tubulin (sc-23948, Santa Cruz, diluted 1:2000). The following secondary antibodies were used: goat anti-rabbit IgG, HRP (ab6721, Abcam, diluted 1:10,000), goat anti-mouse IgG, HRP (AP130P, Sigma, diluted 1:10,000), mouse IgG AF790 (ab175782, Abcam, diluted 1:10,000) and rabbit IgG AF680 (ab175772, Abcam, diluted 1:10,000). Immunoblots were visualised with chemiluminescence for HRP-linked secondary antibodies (Clarity™ Western ECL Substrate, 1,705,060, BioRad) or with fluorescence for the Alexa Fluor labelled secondary antibodies.

### Serum analysis

Commercially available kits were used for the analysis of serum FGF21 (MF2100, R&D systems), GDF15 (MGD150, R&D systems), NEFA (434-91795 and 436-91995, NEFA-HR2 Wako), Triglycerides (290-63701, LabAssay Triglyceride, GPO-DAOS method, Wako), Glycerol (10010755, Cayman Chemical), Insulin (90,080, Crystal Chem) and Leptin (MOB00B, R&D systems) according to the manufacturer’s recommendations.

### Histology

After dissection, iWAT and gonadal WAT (gWAT) were fixated in a 4% paraformaldehyde solution for 24 h. Samples were then rinsed with and stored in PBS until further processing. Before embedding in paraffin, the following dehydration/incubation steps were taken: (1) 30 min in a 75% ethanol solution, (2) 75 min in a 95% ethanol solution (repeated twice), (3) 60 min in a 100% ethanol solution (repeated three times), (4) 60 min in xylene (repeated twice), 3 times in paraffin (of which one overnight, and the others 60 min). Tissue cuttings of 5 µm thickness were cut and mounted on microscope slides (631-1551, VWR). Slides were then dried for 24 h at 37 °C and stained with Mayer’s Haematoxylin (MHS16, Sigma) and Eosin Yellowish (318906, Sigma).

### Statistical analysis

Statistical analysis was performed using GraphPad Prism 9 (GraphPad Software, San Diego, CA USA). A student’s *t*-test (unpaired, 2-tailed) was used to determine differences between WT and UCP1 KO mice within one sex. Two-way ANOVA with factors ‘genotype’ (WT and UCP1 KO) and ‘sex’ (male and female) were used to determine differences between WT and UCP1 KO mice within and between both sexes with multiple comparisons using Tukey’s HSD post hoc test. Repeated-measures ANOVA was used to determine differences between genotypes regarding body weight and fat mass development over time. *P* values of ˂0.05 were considered significant. All graphs display the mean ± SEM.

## Supplementary Information

Supplementary Figures.

## Data Availability

The original contributions presented in the study are included in the article. Further inquiries can be directed to the corresponding author.
