# Supplementary material for: Hyperphagia of female UCP1-deficient mice blunts anti-obesity effects of FGF21
Source: Sci Rep. 2023 Jun 24;13:10288. doi: 10.1038/s41598-023-37264-0 (PMC10290677; doi:10.1038/s41598-023-37264-0)

Supplemental material

Hyperphagia of female UCP1-deficient mice blunts anti-obesity effects of FGF21

Marlou Klein Hazebroek[1], Rutger Laterveer[1], Maria Kutschke[1], Vida Ramšak Marčeta[1], Clarissa S. Barthem[1], Susanne Keipert[1, #]

Supplemental Figure 1

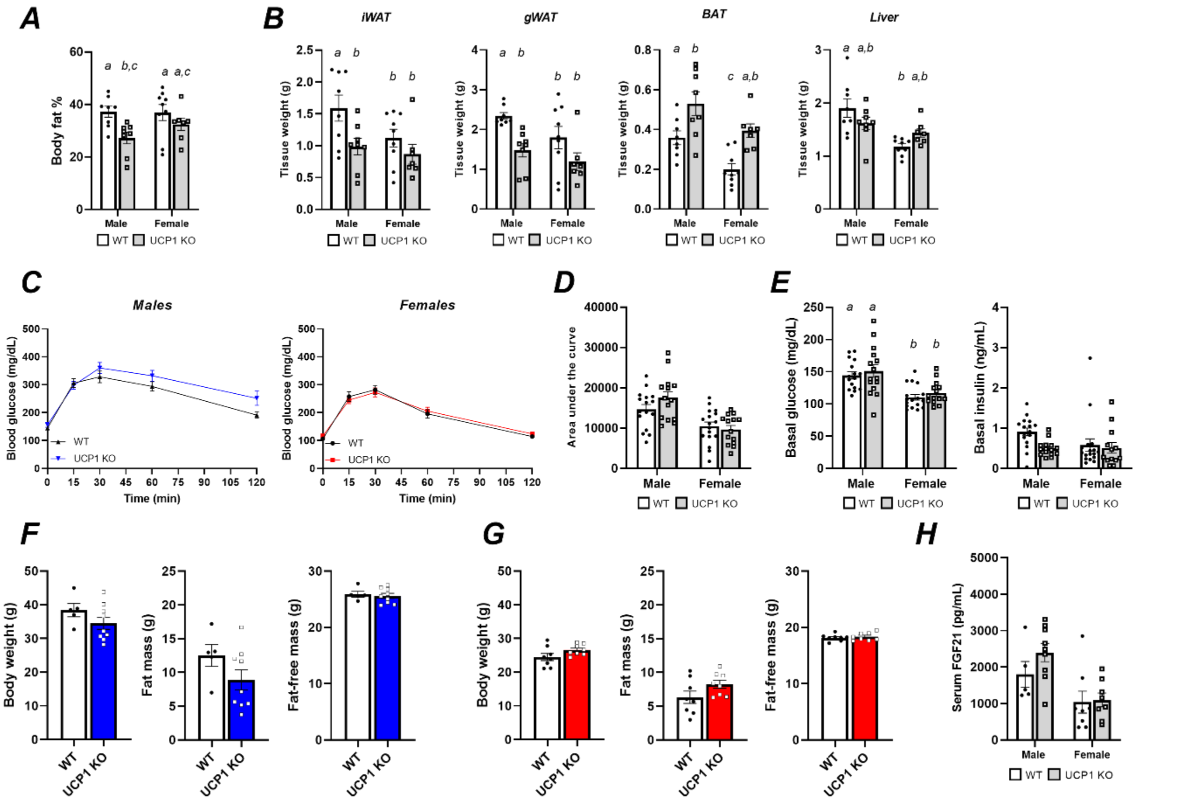

Figure S1. (**A**) Body fat percentage of male and female mice after 15 weeks of HFD at 18°C. (**B**) Tissue weights of iWAT, gWAT, BAT and liver of male and female mice after 15 weeks of HFD. (**C**) GTT after 4 weeks of HFD feeding (after 6h of food withdrawal) of both male and female mice, (**D**) and related area-under-the-curve values. (**E**) Basal glucose and insulin levels at T0 of the GTT after 4 weeks HFD and 6h food withdrawal. (**F**) Final body weight, fat mass and fat-free mass of male (**G**) and female mice kept at 30°C on HFD for 5 weeks. (**H**) Circulating levels of FGF21 of male and female mice after 5 weeks of HFD feeding at 30°C. Figure S1A-B represents *n* = 7-9. Figure S1C-E represents *n* = 15-17. Figure S1F-H represents *n* = 5-9. Significant differences are indicated with different letters (P < 0.05).

Supplemental Figure 2

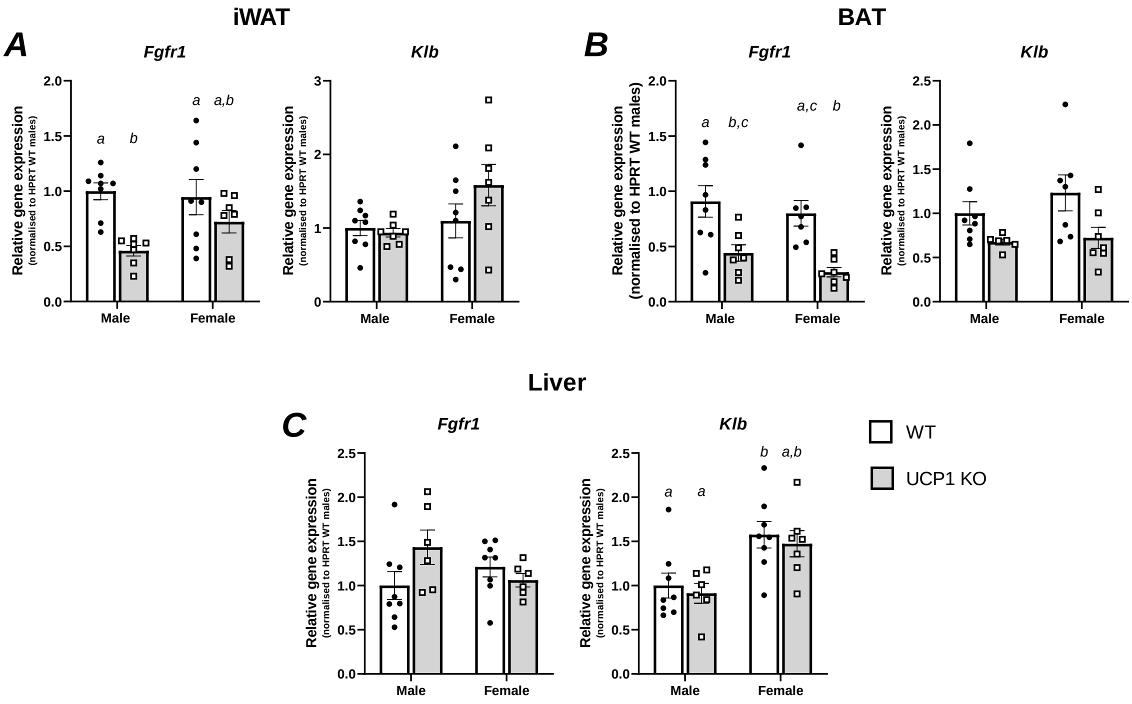

Figure S2. Gene expression of FGF21 receptor 1 (*Fgfr1*) and Beta-Klotho (*Klb*) in male and female mice after 5 weeks of HFD (normalized to the male WT group) in (**A**) iWAT (**B**), BAT (**C**) and liver. All figures represent n = 6-8. Significant differences are indicated with different letters (P < 0.05).

Supplemental Figure 3

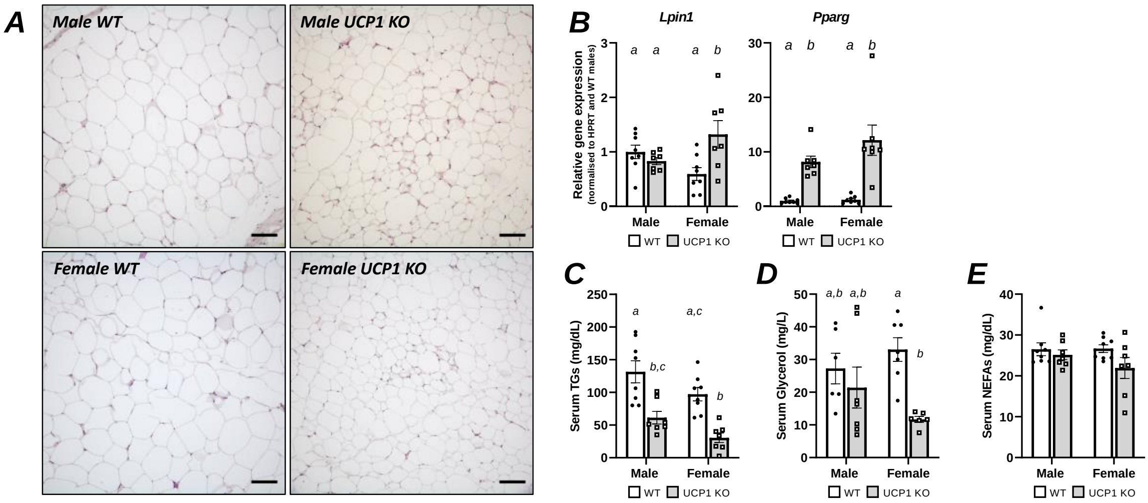

Figure S3. (**A**) Haematoxylin and Eosin staining of iWAT sections of male WT, male UCP1 KO, female WT and female UCP1 KO mice after 15 weeks of HFD. The scale bar indicates 100µm. (**B**) Gene expression from iWAT of genes related to lipid cycling (*Lpin1*) and thermogenic induction (*Pparg*) in male and female mice after 5 weeks of HFD (normalized to the male WT group). (**C**) Serum triglycerides (TGs), (**D**) glycerol (**E**) and non-esterified fatty acids (NEFAs) in male and female mice after 5 weeks of HFD. Figure S3A shows the best representative image of the group (of which 4 animals were analysed). Figure S3B-D represents *n* = 7-8. Significant differences are indicated with different letters (P < 0.05).

Supplemental Figure 4

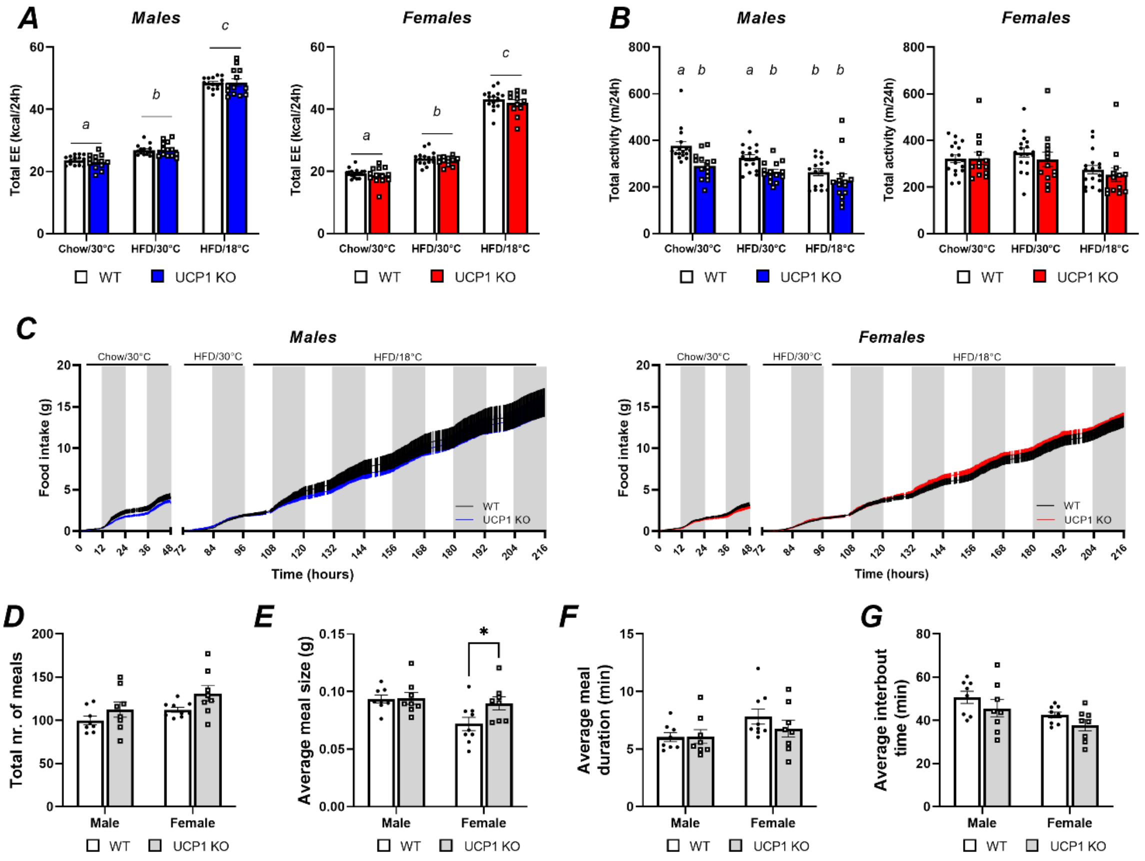

Figure S4. (**A**) Total energy expenditure of males and females over 24h measurement periods during chow feeding at 30°C (0-24h), HFD feeding at 30°C (72-96h) and HFD feeding at 18°C (168-192h). (**B**) Total activity of males and females over 24h measurement periods during chow feeding at 30°C, HFD feeding at 30°C and HFD feeding at 18°C. (**C**) Accumulative food intake of males and females over the complete measurement period (216h), covering chow feeding at 30°C, HFD feeding at 30°C and HFD feeding at 18°C. Detailed analysis of food intake patterns with (**D**) total number of meals, (**E**) average meal size, (**F**) average meal duration and (**G**) average interbout time of animals at 5 weeks of HFD at 18°C. Figures S4A-C represent n = 12-17. Figures S4D-G represent n = 8-9. Significant differences are indicated with different letters ($P < 0.05$) or stars (*$P < 0.05$).

Supplemental Figure 5

Source data (Tyroxine Hydroxylase) for Fig. 2C

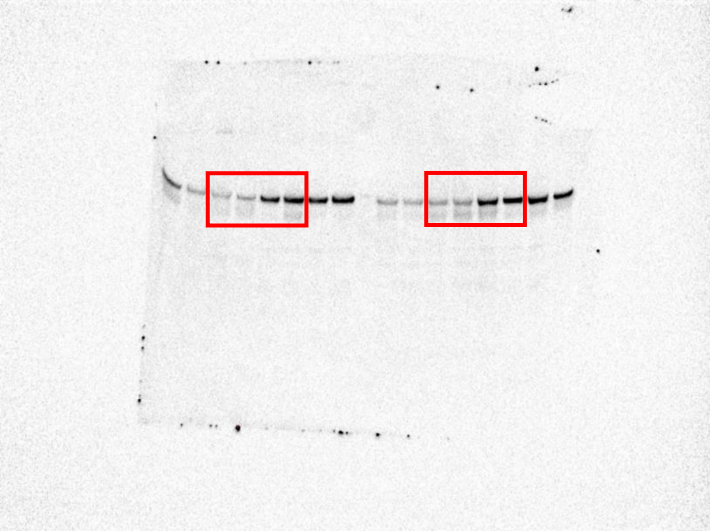

Source data (Alpha-Tubulin) for Fig. 2C

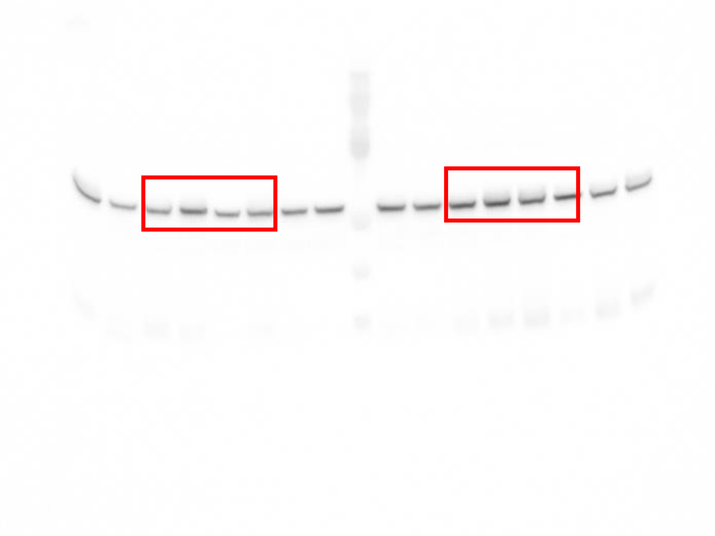

Source data (Glycerol Kinase) for Fig. 3B

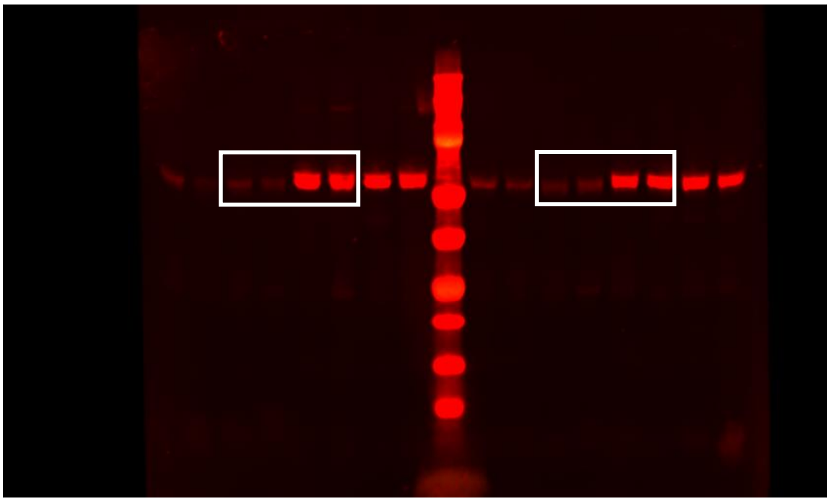

Source data (Total protein – Ponceau staining) for Fig. 3B

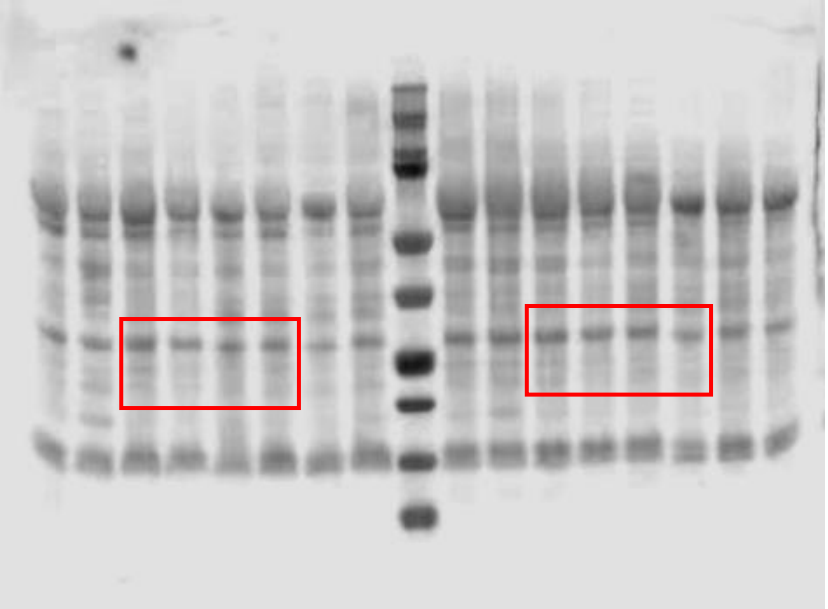

Source data (Glycerol Kinase) for Fig. 3D

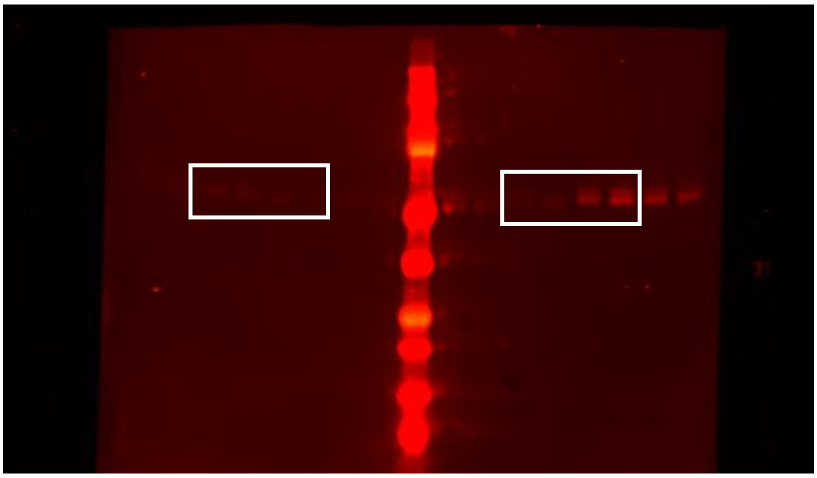

Source data (Alpha-tubulin) for Fig. 3D

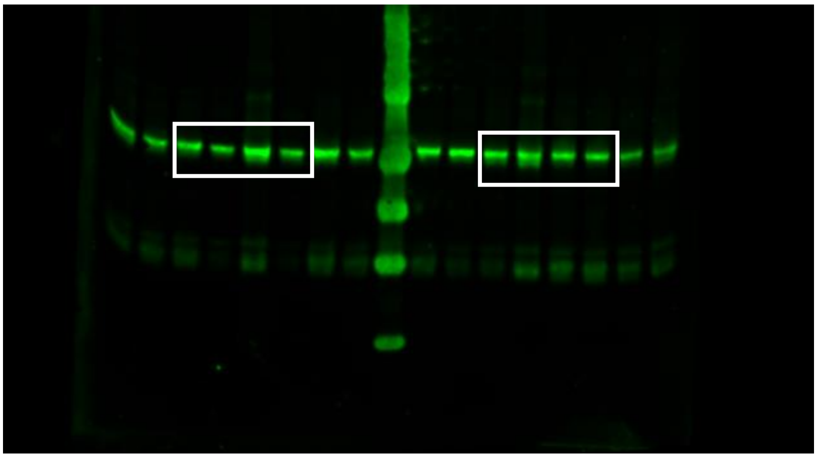

Supplement: Supplementary file 1 — Supplementary Figures. [file 41598_2023_37264_MOESM1_ESM.pdf]
